# Utilizing artificial intelligence-based eye tracking technology for screening ADHD symptoms in children

**DOI:** 10.3389/fpsyt.2023.1260031

**Published:** 2023-11-14

**Authors:** Xiaolu Chen, Sihan Wang, Xiaowen Yang, Chunmei Yu, Fang Ni, Jie Yang, Yu Tian, Jiucai Ye, Hao Liu, Rong Luo

**Affiliations:** ^1^Key Laboratory of Development and Maternal and Child Diseases of Sichuan Province, Department of Pediatrics, Sichuan University, Chengdu, China; ^2^NeuroWeave, Co., Ltd., Shanghai, China

**Keywords:** ADHD, AI eye-tracking technology, intrusive saccades, prosaccade, antisaccade

## Abstract

**Objective:**

To explore the potential of using artificial intelligence (AI)-based eye tracking technology on a tablet for screening Attention-deficit/hyperactivity disorder (ADHD) symptoms in children.

**Methods:**

We recruited 112 children diagnosed with ADHD (ADHD group; mean age: 9.40 ± 1.70 years old) and 325 typically developing children (TD group; mean age: 9.45 ± 1.59 years old). We designed a data-driven end-to-end convolutional neural network appearance-based model to predict eye gaze to permit eye-tracking under low resolution and sampling rates. The participants then completed the eye tracking task on a tablet, which consisted of a simple fixation task as well as 14 prosaccade (looking toward target) and 14 antisaccade (looking away from target) trials, measuring attention and inhibition, respectively.

**Results:**

Two-way MANOVA analyses demonstrated that diagnosis and age had significant effects on performance on the fixation task [diagnosis: *F*_(2, 432)_ = 8.231, ^***^p < 0.001; Wilks’ Λ = 0.963; age: *F*_(2, 432)_ = 3.999, *p < 0.019; Wilks’ Λ = 0.982], prosaccade task [age: *F*_(16, 418)_ = 3.847, ^***^p < 0.001; Wilks’ Λ = 0.872], and antisaccade task [diagnosis: *F*_(16, 418)_ = 1.738, *p = 0.038; Wilks’ Λ = 0.938; age: *F*_(16, 418)_ = 4.508, ^***^p < 0.001; Wilks’ Λ = 0.853]. Correlational analyses revealed that participants with higher SNAP-IV score were more likely to have shorter fixation duration and more fixation intervals (*r* = −0.160, 95% CI [0.250, 0.067], ^***^p < 0.001), poorer scores on adjusted prosaccade accuracy, and poorer scores on antisaccade accuracy (Accuracy: *r* = −0.105, 95% CI [−0.197, −0.011], *p = 0.029; Adjusted accuracy: *r* = −0.108, 95% CI [−0.200, −0.015], *p = 0.024).

**Conclusion:**

Our AI-based eye tracking technology implemented on a tablet could reliably discriminate eye movements of the TD group and the ADHD group, providing a potential solution for ADHD screening outside of clinical settings.

## Introduction

Attention-deficit/hyperactivity disorder (ADHD) is a neurodevelopmental disorder characterized by persistent and age-inappropriate inattention, hyperactivity, and/or impulsivity. The prevalence of ADHD is increasing, with rates of 7.2% globally and 6.26% in China (1, 2). The prevalence in China is likely an underestimation due to many unreported cases as there is a shortage of specialized pediatric psychiatrists, especially in rural areas. As ADHD is a chronic disorder that has a significant impact on the individual, their family, and society, it is critical to screen and diagnose ADHD accurately in order to provide early intervention for children with ADHD (3).

The current diagnostic process for ADHD within the clinical setting primarily relies on a subjective interview and standardized rating scales (4, 5). Although P300 event-related potentials are promising electroencephalography signatures that can objectively discriminate individuals with ADHD, it is not formally used for screening or diagnostic purposes due to its need for sophisticated equipment (6, 7). Other physiological differences have been reported on the markers within the heart–brain and gut–brain axes as well as motor cortex physiology; however, these remain correlative measures and are unable to provide diagnostic confirmation (8, 9). Given the complex etiology of ADHD and the common occurrence of multiple comorbidities, it is therefore challenging to identify children with ADHD by the present procedures, which are based on subjective assessments. This highlights the need for objective and quantifiable neurobehavioral tests and measures that can more quickly and more accurately facilitate diagnostic screening methods for earlier interventions (4, 5, 10).

With the advancement in eye tracking technology, voluntary and involuntary eye movements can be registered to assess ocular dysfunction and neural mechanisms underlying attention, emotions, and intentions (11). A commonly tracked parameter is saccades, which are eye movements involving the sequential alignment of the fovea toward objects of interest in the periphery, through swift and discrete step-like movements. Eye tracking experiments have been shown to be useful for the diagnosis of neuropsychiatric and neurological disorders, including bipolar disorder, mild cognitive impairment, Alzheimer’s disease, Parkinson’s disease, autism, and ADHD (12). Particularly, children with ADHD have a greater number of intrusive saccades during the eye fixation task, with a shorter antisaccade latency compared to children without ADHD (13, 14). Children with ADHD are also more likely to make directional errors and have slower saccade reaction times during an antisaccade task, during which the eye movement should be opposite to the visual stimuli.

Although eye tracking technology can be a useful clinical tool to guide diagnosis, it typically requires the use of specialized equipment, including a chin and head rest, a high-speed camera, and an additional computer screen (15, 16). This limits its use outside of a research lab and thus is not ideal as a clinical tool. As such, the present study aimed to determine whether our artificial intelligence (AI)-based eye tracking technology, which only requires a tablet, can be used as a tool to screen for ADHD symptoms in children aged 6–12 years old.

## Materials and methods

### Participants

We recruited 639 children in grades 1–6 at the Second Primary School in Wanyuan City for this study, and 437 participants who fulfilled the inclusion criteria were enrolled in this study. Although there were no drop outs, 43 were omitted as they did not complete the eye-tracking task successfully. The inclusion criteria were as follows: aged 6–12 years old, consented to undergo assessment using the criteria described in the Diagnostic and Statistical Manual of Mental Disorders, Fifth Edition (DSM-5), and completed at least three prosaccade and three antisaccade trials.

The exclusion criteria were as follows: current, controlled (on medication) or uncontrolled, comorbid psychiatric diagnosis; currently diagnosed with mental retardation [IQ < 80 determined using the Wechsler Intelligence Scale for Children (WISC-IV)]; eye movement disorder; history of seizures, or significant motor or vocal tics, including but not limited to Tourette’s disorder; uncorrected refractive error and distance visual acuity; diagnosis of or parent-reported color blindness, which would prevent independent completion of the eye tracking test. The enrolled participants were then broadly divided into the ADHD and typically developing (TD) groups based on the DSM-5 diagnostic criteria, which are further elaborated in the data analysis section below.

### Apparatus

The task does not require the screen resolution to be specifically high, and most consumer-grade portable devices will be sufficient to carry out the task. We used a Lenovo^®^ Yoga Tab 13 Tablet without additional sensors or devices to record the eye movements, eye gaze, and head positions of the participants at 30 Hz. We designed a data-driven end-to-end convolutional neural network appearance-based model that uses the information from serial images to robustly predict eye gaze, with an accuracy of 2 cm based on the average Euclidean distance error from the true fixation location.

To ensure the AI model was robust in predicting eye gaze, we used the Gaze Capture Dataset to train our AI model (17). This dataset features a sizable compilation of eye-tracking data derived from over 1450 individuals and encompasses close to 2.5 million images. Due to the diversity of the dataset, the AI model is adapted to faces of most ethnicities. We performed an initial experiment collecting data from 258 participants, who were requested to sequentially shift their focus between focal points displayed on a screen. Over 232,200 frames were collected and used to analyze the corresponding relation between images of faces, eyes, and gaze location. The accuracy was determined using the average Euclidean distance error, which was found to be less than 2 cm. This indicates that the predicted gaze point could potentially lie up to 2 cm away from the actual fixation location.

Using a distributed processing model, data were collected and uploaded to the cloud server for processing and subsequently returned (Supplementary Figure 1). This procedure allows the tablet to be the sole data collection terminal, thus reducing the resources required for the eye tracking test.

### Data processing

The recordings of eye movements were sent to the cloud, where the server acts as a data warehouse and a center for data processing. Parameters including latency, velocity, gain, amplitude, fixation duration, and error rate for cognitive tasks were detected via AI algorithmic calculations.

We have previously implemented the use of an appearance-based model that requires a large amount of data but generalizes well to novel faces. It uses frames with both the face and eyes for detection as crucial inputs, such that eye tracking can only operate if the whole face is visible. The coordinates of the left eye, right eye, and face are then entered as a 1 × 6 vector (Supplementary Figure 2). As long as the face can be captured in the center of the screen without any obstruction, the model would adjust to fit to the size of the face.

By using an appearance-based model, we could achieve end-to-end matching by constructing a sample based on feature recognition, which can tolerate a relatively low resolution and the sample rate of the front-facing camera of the tablet. To calibrate the eye tracker, the subject will be required to stare at the center fixation point on the screen for 500 ms before each trial, minimizing the need for a tedious calibration process that is required for traditional eye tracking technologies.

### Stimuli used in the eye tracking test

The stimulus used in the fixation task is a dot measuring 25 pixels. This value is the same diameter as the dot size described by Bucci. et al. (18) with an RGB value of [75, 35, 35] presented on a light-yellow screen with an RGB value of [252, 248, 230]. We used the following formula of arc length to calculate the size of the stimulation dot:


L=ω×r

$$=22.2^{{}^{\circ}}\times 2\,\mathrm{pixel}/720\times 60\,\mathrm{cm}$$

=5.236⁢mm

where ω is the angle in radians, and r is the radius, 60 cm being the average relative distance from the eye to the screen based repeated trials. Based on the dimensions of the Yoga 13 tablet, which are 293.4 mm × 204 mm, the size of the dot will be:


=1440×5.236/293.4=25.698⁢pixels

As such, we have selected a dot size of 25 pixels for convenient calculation and programming, which will be located at the center of the screen for a duration of 30 s (Supplementary Figure 3).

In the saccade task, the stimulus will appear on either the right or the left side of the screen with a point-size of 0.5 degrees (18). The distance between the stimulus and the initial instructional dot was calculated as follows:


(Number⁢of⁢pixels⁢along⁢width×arc⁢length/screen⁢length)/2

=(1440×5.236⁢cm/293.4⁢mm)/2

=570⁢pixels

To minimize the contrast between the background and the stimuli (dots and crosses), we opted against using a pure black RGB [0, 0, 0] and instead used a yellowish hue [252, 248, 230] to provide a soft contrast and a more user-friendly viewing experience over an extended period, if necessary. To minimize potential confounding variables, we utilized the most prominent colors (19) that were used in previous studies—red [255, 0, 0] and green [0, 255, 0]—to indicate antisaccade and prosaccade trials, respectively.

### Eye tracking test (18)

The participants were instructed to not consume any medications for ADHD treatment (if any) prior to the eye tracking test in order to minimize confounding effects. This was confirmed by verifying with the child’s guardian prior to the task. During the eye tracking test, the participants were seated 60–80 cm away from the screen in a well-lit room (between 800 and 1200 lux) (20) and instructed by the research staff to keep their head and body as still as possible. Visual stimuli were delivered via the tablet with a 13-inch display and a resolution of 1440 × 900. The viewing distance was adjusted by the participant until both eyebrows and the chin were clearly visible on the screen. The participants then completed an eye fixation task, 14 prosaccade trials, and 14 antisaccade trials during the test.

Calibration was performed at the beginning of each trial, during which the participants were required to fixate on the center cross for a minimum of 500 ms. During the eye fixation task, the participants must maintain their eye gaze on the target, which was 30 s long. The participants then had to complete 14 prosaccade and 14 antisaccade trials (presented randomly), which took about 5 min. The whole process should be completed in about 6 min. Only participants who completed at least three prosaccade and three antisaccade trials were included in this study.

During the prosaccade and antisaccade trials, the participants were presented first with the current trial number and the number of subsequent trials to be followed in the center of the screen for 1000 ms (Supplementary Figure 2). Following that, a central cross appeared at the center of the screen for a period of 1500–2500 ms. This was followed by a 200-ms gap, when the central cross disappeared. A central fixation point then reappeared with a target on either the left or right of the fixation point for 3000 ms. A green central fixation point indicated a prosaccade trial, and the participants needed to quickly shift their gaze to the target. A red central fixation point indicated an antisaccade trial, and the participants needed to shift their gaze opposite of the target. After the central fixation point and target disappeared, a blank screen appeared for 1000 ms before the next trial was presented with the next trial number. The presentation of the prosaccade and antisaccade trials as well as the lateral side of the target were randomized.

A successful trial was characterized by the collection of reliable and valid data by the camera, which could then be subjected to AI-based analysis. Certain trials may have failed due to various reasons. For instance, the whole face may not have been completely captured by the camera, so the model could not register the person’s facial features. Participants who failed to maintain eye contact with the fixation point during the initial calibration period would also result in the model not being able to calculate eye-tracking points. Similarly, when the tablet was moved without the teacher’s consent and exposed to outdoor bright lighting, the face in the foreground would become too dark to be recognized by the detection algorithm. As such, we established a criterion of having to complete three of each trial type to be included in the study, which would account for approximately 20% of the 14-trial session.

### Definitions of parameters measured

The parameters measured during the eye tracking test and their definitions are presented in Table 1. Briefly, these parameters measure how fast the participants respond as well as their attention, accuracy of performance, and ability to correct erroneous trials.

**TABLE 1:** The parameters measured during the eye tracking test and their definitions.

Parameter	Definition
Accuracy	Percentage of accurately shifting eye gazes toward the stimuli in prosaccade trials and shift away from the stimuli in antisaccade trials.
Adjusted accuracy	The sum of the percentage of successful initial shifts toward the eye gaze and the percentage of incorrectly directed initial saccades that were subsequently rectified in the correct direction within a trial.
Amplitude	Size of the saccade, measured in pixels or degrees of arc. A small amplitude indicated that the eye had travelled a shorter distance during a saccade
Central area fixation duration	Time spent in the central area (± 100 ms from the center of the screen) during a simple fixation task
Correction latency	Time taken for an erroneous saccade
Fixation interval	Number of saccades during a simple fixation task
Gain	Ratio of the actual amplitude of the saccade to the expected amplitude based on the distance between the central fixation point and the target
Latency	Time taken from the appearance of a target to the beginning of a saccade in response to a target
Peak velocity	Highest velocity achieved during the onset of a saccade
Velocity	Amplitude of the saccade divided by the duration of the saccade, reported in pixels per ms

### Data analysis

The participants were first stratified into two groups: TD group and children with ADHD (ADHD group). The participants were sorted into the TD group if they did not meet the diagnostic criteria for ADHD based on DSM-5.

We used GraphPad Prism^®^ software (V8.0.0 for Mac, GraphPad Software, San Diego, CA, USA) and JASP to perform the statistical analyses. Two-way multivariate analysis of variance (MANOVA) were performed, whereby the participants were grouped based on two independent variables [diagnosis: TD or ADHD; and age: <10 (“young” group) or ≥10 years old (“older” group)]. We used central tendency measures to analyze the data. Dependent variables including the saccade latency and velocity were then analyzed for both the antisaccade and prosaccade trials according to the independent variables. The fixation period and the time spent in the center of the screen were also analyzed. If the main effect of a dependent variable was significant, we performed *post-hoc* analyses using the Tukey’s-Kramer test. If the interaction was significant, we followed up with a simple effect analysis. A *p*-value < 0.05 was considered statistically significant.

## Results

### Participant demographics

The 437 participants enrolled into this study were divided into two groups: the TD group, who did not meet the diagnostic criteria for ADHD based on DSM-5 (*n* = 325), and the ADHD group (*n* = 112) (Table 2). All parameters measured in the fixation, prosaccade and antisaccade tasks are summarized in Table 3.

**TABLE 2:** Demographic characteristics of the participants.

	TD	ADHD
Number of subjects	325	112
Female (%)	21.85	28.57
Age (years)	9.45 ± 1.59	9.40 ± 1.70
SNAP-IV score (mean ± SD)	8.23 ± 7.89	28 ± 7.64

**TABLE 3:** Parameters measured during the eye tracking task, stratified according to TD or ADHD.

	TD (*n* = 325)	ADHD (*n* = 112)
**Fixation task**		
Central fixation duration (ms)	24408.30 ± 7351.23	21614.85 ± 8999.36
Fixation interval	10.84 ± 8.18	13.43 ± 9.08
**Prosaccade task**		
Latency (median, ms)	342.80 ± 90.23	334.89 ± 96.66
Latency (mean, ms)	366.17 ± 100.16	363.69 ± 114.40
Latency (correct median, ms)	341.73 ± 98.53	332.25 ± 97.11
Latency (correct mean, ms)	366.44 ± 105.01	355.18 ± 119.95
Velocity (median, pixel/ms)	2.57 ± 0.72	2.56 ± 0.72
Velocity (mean, pixel/ms)	2.66 ± 0.67	2.676 ± 0.69
Velocity (peak median, pixel/ms)	5.71 ± 1.80	5.87 ± 1.87
Velocity (peak mean, pixel/ms)	5.98 ± 1.82	6.19 ± 1.97
Initial amplitude (median)	9.27 ± 3.76	9.67 ± 4.63
Initial amplitude (mean)	12.79 ± 8.63	13.49 ± 9.27
Final gain (median)	0.58 ± 0.18	0.59 ± 0.19
Final gain (mean)	0.69 ± 1.51	0.60 ± 0.20
Correction latency (median, ms)	64.69 ± 69.84	58.80 ± 43.97
Correction latency (Mean, ms)	91.55 ± 74.13	86.44 ± 54.00
Accuracy	61.79 ± 15.05%	59.94 ± 13.67%
Accuracy (adjusted)	97.53 ± 6.64%	96.03 ± 9.18%
**Antisaccade task**		
Latency (median, ms)	328.85 ± 91.07	328.26 ± 98.26
Latency (mean, ms)	357.15 ± 98.53	351.95 ± 112.72
Latency (correct, median, ms)	350.77 ± 131.74	351.07 ± 166.46
Latency (correct, mean, ms)	368.30 ± 131.91	362.70 ± 146.98
Velocity (median, pixel/ms)	2.61 ± 0.70	2.68 ± 0.68
Velocity (mean, pixel/ms)	2.66 ± 0.66	2.74 ± 0.65
Velocity (peak, median, pixel/ms)	6.27 ± 2.19	6.77 ± 2.24
Velocity (peak, mean, pixel/ms)	6.47 ± 2.07	6.91 ± 2.14
Initial amplitude (median)	9.13 ± 3.77	10.82 ± 12.56
Initial amplitude (mean)	13.64 ± 12.79	16.72 ± 21.93
Final gain (median)	0.57 ± 0.18	0.61 ± 0.19
Final gain (mean)	0.59 ± 0.26	0.69 ± 0.78
Correction latency (median, ms)	189.23 ± 154.72	234.41 ± 165.33
Correction latency (mean, ms)	220.52 ± 147.48	260.25 ± 159.47
Accuracy	47.70 ± 16.54%	44.98 ± 15.03%
Accuracy (adjusted)	91.01 ± 13.47%	90.02 ± 14.39%

### Fixation task

To understand how diagnosis and age groups affected performance on the fixation task, we performed two-way MANOVA on the parameters measured during the task. The two-way MANOVA showed a significant multivariate group effect of both diagnosis [*F*_(2, 432)_ = 8.231, ^***^p < 0.001; Wilks’ Λ = 0.963] and age [*F*_(2, 432)_ = 3.999, *p < 0.019; Wilks’ Λ = 0.982] on the overall performance and the ability to maintain attention during the task. However, the interaction between diagnosis and age groups was not significant [*F*_(2, 432)_ = 0.691, *p* = 0.501], suggesting age might be a factor that contribute to performance independent of diagnosis group, and vice versa.

Univariate analyses revealed that diagnosis group had a significant effect on parameters measuring visual steadiness during the fixation task [number of fixation intervals: *F*_(1, 433)_ = 7.822, ^**^p = 0.005, ω^2^ = 0.015; central area fixation duration: *F*_(1, 433)_ = 10.82, ^**^p = 0.001, ω^2^ = 0.022]. However, age group only had a significant effect on the parameters measuring fixation duration on the central area [the number of fixation intervals: *p* = 0.244; central area fixation duration: *F*_(1, 433)_ = 4.565, *p = 0.033, ω^2^ = 0.008]. This indicated that the ability to remain on task was dependent on diagnosis group, while the ability to maintain attention was dependent on both diagnosis and age groups.

**FIGURE 1:**
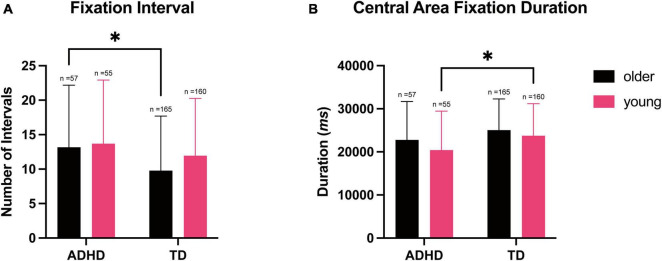
Number of intervals and central area fixation duration during simple eye fixation tasks, stratified according to the diagnosis and age. Data shown are expressed as the mean ± SD, *n* = 437. **(A)** Two-way ANOVA showed the main effect of the diagnosis on the number of intervals compared to the TD group; the ADHD group performed significantly more saccades in the older participants, *p = 0.017. **(B)** Two-way ANOVA showed the main effect of diagnosis and age on the central area fixation duration; the younger participants with ADHD spent significantly less time on the central area than the TD group, *p = 0.031.

*Post-hoc* Tukey’s test on the number of fixation intervals showed that the ADHD group exhibited a greater occurrence of saccades compared to the TD group during the fixation task (*Cohen’s d* = 0.306, 95% CI [0.090, 0.523], ^**^p = 0.005, Figure 1A). The TD group also had a longer central area fixation duration than the ADHD group (*Cohen’s d* = 0.361, 95% CI [0.144, 0.577], ^***^p < 0.001, Figure 1B), while younger participants also had shorter central area fixation duration (*Cohen’s d* = - 0.234, 95% CI [−0.450, −0.018], *p = 0.033, Figure 1B).

To further understand the relationship between diagnosis and age groups on fixation task performance, we performed correlation analysis and found that participants with higher SNAP-IV score were more likely to have a shorter central fixation duration and higher number of fixation intervals (*r* = −0.160, 95% CI [0.250, 0.067], ^***^p < 0.001). Using diagnosis group as a factor and age as a covariate, we constructed a regression model using central area fixation duration or the number of fixation intervals as dependent variables, TD participants would have an average of 2766.8 ms (adjusted *r*^2^ = 0.033, 95% CI [1095.3, 4438], ^**^p = 001) longer duration on the central area compared to participants with ADHD. Similarly, for every year increase in age would predict an average of 562.6 ms (95% CI [109.5, 1016], *p = 0.015) increase in central area fixation duration.

### Prosaccade task

Two-way MANOVA showed a significant multivariate effect of age group on the performance on prosaccade task, which mainly measured the responses to reflexive eye movements [age group: *F*_(16, 418)_ = 3.847, ^***^p < 0.001; Wilks’ Λ = 0.872]. Similar to the fixation task, there was no significant interaction between diagnosis and age groups [Diagnosis group *F*_(16, 418)_ = 1.103, *p* = 0.350, Wilks’ Λ = 0.959, interaction: *F*_(16, 418)_ = 1.393, *p* = 0.141, Wilks’ Λ = 0.949].

Univariate analyses revealed that age had a significant effect on the parameters measured in the prosaccade task [mean of saccade latency: *F*_(1, 433)_ = 19.01, ^***^p < 0.001, ω^2^ = 0.040; median of saccade latency: *F*_(1, 433)_ = 18.56, ^***^p < 0.001, ω^2^ = 0.039; adjusted accuracy: *F*_(1, 433)_ = 7.488, ^**^p = 0.006, ω^2^ = 0.015; median of peak velocity: *F*_(1, 433)_ = 6.770, ^**^p = 0.010, ω^2^ = 0.013]. Diagnosis group, however, only had a marginal significant effect on the adjusted prosaccade accuracy [adjusted accuracy: *F*_(1, 433)_ = 3.551, *p* = 0.060, ω^2^ = 0.006]. This suggested that performance on the prosaccade task was dependent on age.

**FIGURE 2:**
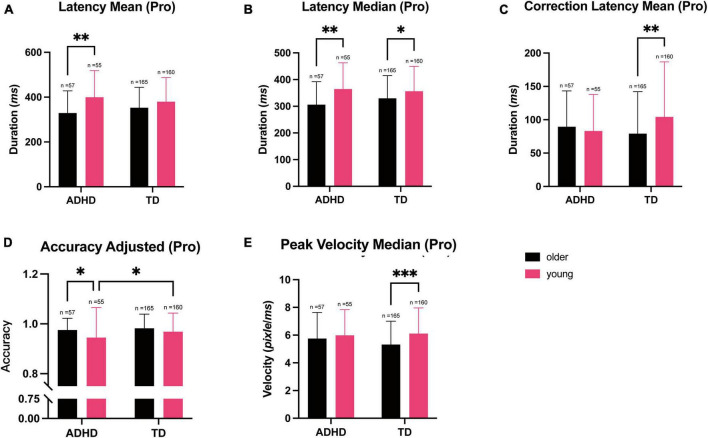
The parameters measured during the prosaccade trials during the eye tracking test and stratified according to the diagnosis and age. Data are represented as the mean ± SD, *n* = 437. **(A)** Two-way ANOVA showed a longer mean (*p = 0.019) and **(B)** median (*p = 0.019) latency in the younger participants than in the older participants in the ADHD group. **(C)** The mean (**p = 0.007) correction latency was longer in the younger participants compared to the older participants in the TD group. **(D)** The adjusted accuracy was lower in the ADHD participants in the younger group compared to the older group (*p = 0.028), while among the TD participants, the younger group had a lower adjusted accuracy than the older group (*p = 0.039). **(E)** The median peak velocity was higher in the younger participants compared to the older participants in the TD group (***p < 0.001).

*Post-hoc* Tukey’s test showed that younger participants exhibited slower saccades compare to the older participants (mean of prosaccade latency: *Cohen’s d* = 0.478, 95% CI [0.260, 0.696], ^***^p < 0.001; Figure 2A). Similarly, younger participants also exhibited slower saccades compared to the older participants (median of prosaccade latency: Cohen’s d = 0.472, 95% CI [0.254, 0.690], ^***^p < 0.001, Figure 2B). *Post-hoc* Tukey’s test on the mean prosaccade correction latency also demonstrated that the older participants in the TD group had a shorter median correction latency compared to the younger participants (*Cohen’s d* = −0.362, 95% CI [−0.658, −0.066], ^**^p = 0.007). While older participants had a longer median correction latency compared to the younger participants within the ADHD group, this difference was non-significant (*Cohen’s d* = 0.092, 95% CI [−0.410, 0.593]; *p* = 0.963, Figure 2C).

When comparing adjusted accuracy, adjusted accuracy was higher in the older group compared to the younger ground (*Cohen’s d* = 0.300, 95% CI [0.084, 0.516], ^**^p = 0.006 Figure 2D), and was lower in the ADHD group compared to the TD group (*Cohen’s d* = −0.206, 95% CI [−0.422, 0.009], *p* = 0.060, Figure 2D). When comparing peak velocity on the task, the younger group had faster saccades compared to the older group (median: *Cohen’s d* = 0.285, 95% CI [0.069, 0.501], *p = 0.010, Figure 2E).

We then performed correlation analysis using the parameters measured in the prosaccade task and SNAP-IV score, and found that participants with a higher SNAP-IV score had poorer scores on the adjusted prosaccade accuracy (*r* = −0.120, 95% CI [−0.211, −0.026], *p = 0.012), while the other parameters did not have significant correlations. Using diagnosis group as a factor, age as a covariate and adjusted prosaccade accuracy as a dependent variable, TD subjects would have a predicted higher accuracy of an average 1.5% (adjusted *r*^2^ = 0.030, 95% CI [−0.1, 3%]) on the prosaccade task compared to the ADHD group (*p* = 0.068). For every year increase in age would predict an average of 0.8% increase (95% CI [0.3, 1.2%], ^***^p < 0.001) in adjusted accuracy. This indicated that adjusted accuracy on the prosaccade task is more dependent on age.

### Antisaccade task

Two-way MANOVA revealed a significant multivariate effect of both diagnosis and age groups on the performance on the antisaccade task, which test for voluntary eye movement response and the inhibition abilities [age group: *F*_(16, 418)_ = 4.508, ^***^p < 0.001; Wilks’ Λ = 0.853], diagnosis group [*F*_(16, 418)_ = 1.738, *p = 0.038; Wilks’ Λ = 0.938]. The interaction between diagnosis and age group was also significant [*F*_(16, 418)_ = 4.508, *p = 0.027; Wilks’ Λ = 0.935].

Univariate analysis showed that there was a significant effect of age on the parameters measured on the antisaccade task [Adjusted accuracy: *F*_(1, 433)_ = 3.926, *p = 0.048, ω^2^ = 0.007]; Mean of correction latency: [*F*_(1, 433)_ = 15.50, ^***^p < 0.001, ω^2^ = 0.031]; Median of correction latency: [*F*_(1, 433)_ = 11.71, ^***^p < 0.001, ω^2^ = 0.023]. Diagnosis group similarly had a significant effect on the parameters Median of Initial amplitude: [*F*_(1, 433)_ = 4.758, *p = 0.030, ω^2^ = 0.009]; Median of peak velocity: [*F*_(1, 433)_ = 4.186, *p = 0.041, ω^2^ = 0.007]; Mean of correction latency: [*F*_(1, 433)_ = 6.089, *p = 0.014, ω^2^ = 0.011]; Median of correction latency: [*F*_(1, 433)_ = 7.138, ^**^p = 0.008, ω^2^ = 0.013].

**FIGURE 3:**
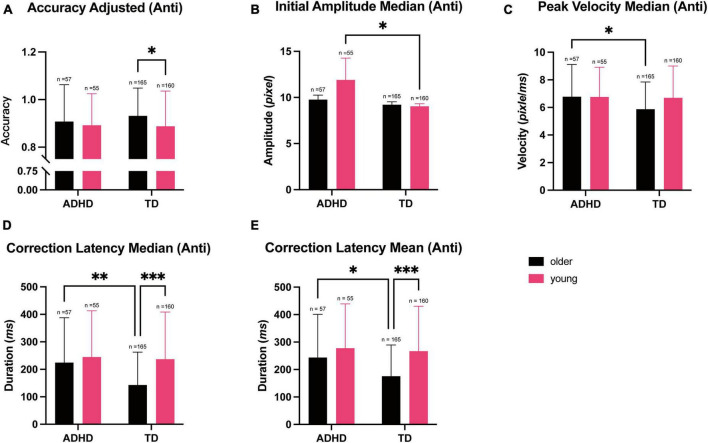
Parameters measured during the antisaccade trials during the eye tracking test and stratified according to the diagnosis and age. Data are represented as the mean ± SD, *n* = 437. **(A)** The adjusted accuracy was lower in the younger participants in the TD group compared to the older group (*p = 0.020). **(B)** The median initial amplitude was higher in the ADHD group compared to the TD group among the younger participants (*p = 0.010). **(C)** The median peak velocity was higher in the ADHD participants compared to the TD participants in the older group (*p = 0.406). **(D)** The median correction latency was higher in the ADHD group compared to the TD group (**p = 0.003). The median correction latency was also higher in the younger participants compared to the older participants (***p < 0.001). **(E)** The mean correction latency was higher in the ADHD group compared to the TD group (*p = 0.014). The mean correction latency was also higher in the younger participants compared to the older participants (***p < 0.001).

*Post-hoc* Tukey’s test showed that the adjusted accuracy on the antisaccade task was higher in the older group compared to the younger group (*Cohen’s d* = 0.217, 95% CI [0.001, 0.433], *p = 0.048, Figure 3A). Initial amplitude median was higher in the ADHD group compared to the TD group, in the younger participants (*Cohen’s d* = 0.239, 95% CI [0.023, 0.455], *p = 0.030, Figure 3B). Median peak velocity, on the other hand, was higher in the ADHD group compared to the TD group within the older group (*Cohen’s d* = *0.224*, 95% CI [0.008, 0.440], *p = 0.041, Figure 3C). However, median correction latency was higher in the ADHD group compared to the TD group (median: *Cohen’s d* = 0.293, 95% CI [0.077, 0.509], ^**^p = 0.008, Figure 3D), indicating that the ADHD group required more time to detect and correct errors. Younger participants also required more time to correct errors compared to the older participants (median: *Cohen’s d* = 0.375, 95% CI [0.158, 0.592], ^***^p < 0.001, Figure 3E).

We then performed correlation analysis using the parameters from the antisaccade task and SNAP-IV score, and found that the initial amplitude and correction latency were positively correlated with SNAP-IV scores (median of initial amplitude: *r* = 0.105, 95% CI [0.011, 0.197], *p = 0.028; Mean of initial amplitude: *r* = 0.094, 95% CI [0.000, 0.186], *p = 0.049; Median of correction latency: *r* = 0.181, 95% CI [0.089, 0.270], ^***^p < 0.001; Mean of correction latency: *r* = 0.146, 95% CI [0.053, 0.147], ^**^p = 0.002). Accuracy on the task, however, was negatively correlated with the SNAP-IV score, indicating that participants higher SNAP-IV were likely to have lower accuracy (Accuracy: *r* = −0.105, 95% CI [−0.197, −0.011], *p = 0.029; Adjusted accuracy: *r* = −0.108, 95% CI [−0.200, −0.015], *p = 0.024).

Using measures that correlated with SNAP-IV score as a variable, we constructed regression models for each measure using diagnosis group as a factor and age as a covariate. We observed that there was a reduction in the median initial amplitude for the TD group compared to the ADHD group by 1.676, while there was a reduction in the mean amplitude of 3.059 for TD group compared to ADHD group (median of initial amplitude: 95% CI [0.140, 3.213], *p = 0.033; mean of initial amplitude: 95% CI [0.312, 6.430], *p = 0.075), These models, however, were not significant (median of initial amplitude: *p* = 0.509; mean of initial amplitude: *p* = 0.497).

There was a reduction in correction latency for every year increase in age by an average of 28.115 in the median, and an average of 28.844 in mean (median of correction latency: 95% CI [19.299, 36.930], ^***^p < 0.001; mean of correction latency: 95% CI [20.470, 37.217], ^***^p < 0.001). There was also an increase in correction latency in the TD group compared to the ADHD group by an average of 43.841 for median, and 38.359 for mean (median of correction latency: 95% CI [11.322, 76.361], ^**^p = 0.008; mean of correction latency: 95% CI [7.471, 69.248], *p = 0.015). These suggested that correction latency depended strongly on both age and diagnosis group.

With every year increase in age, there was also an increase of accuracy by 1.7%, and increase of adjusted accuracy of 1.8% (accuracy: 95% CI [0.8%, 2.7%], ^***^p < 0.001; adjusted accuracy: 95% CI [1.1, 2.6%], ^***^p < 0.001). This was, however, not significant for diagnosis groups (accuracy: *p* = 0.132; adjusted accuracy: *p* = 0.538), indicating that accuracy was more dependent on age.

## Discussion

In this study, TD children and ADHD children completed an eye tracking task comprised of a simple fixation task, prosaccade trials, and antisaccade trials. This was delivered via a tablet with AI software that we had developed, allowing the measurements of attention, reflexive gaze toward a target, and voluntary gaze away from a target that requires inhibitory control, respectively (13, 21). We found that during the simple fixation task, the ADHD participants exhibited more saccades compared to the TD participants, resulting in a lower amount of time spent fixating on the central area. The younger participants in general also had a shorter fixation duration compared to the older participants. During the prosaccade trials of the eye tracking task, age was a contributing factor to the differences in latency and peak velocity. The younger participants had a slower reaction time, although their velocity was faster. The lack of significant main effect of diagnosis and interaction between age and diagnosis for prosaccade trials was unexpected, however, these may require a higher level of sensitivity when detecting differences in performance across diagnosis and age groups.

During the antisaccade trials, the participants with ADHD had higher amplitudes and a slower correction latency, requiring more time to correct errors and redirect saccades to the correct direction. Age similarly contributed to a slower saccade latency in the younger participants. While the correction latency during the prosaccade trials was dependent on the age of the participants, the number of errors made during the prosaccade trials was higher in the younger ADHD group. In addition, we observed that the ADHD group required a longer duration to correct an erroneous saccade compared to the other group during the antisaccade task, and this again was more prominent in the younger cohort. Our present study also found a significant interaction between age and diagnosis in antisaccade trials, demonstrating that antisaccade trials might be robust at discriminating between ADHD and TD groups, while also detecting developmental changes across age. This thus provides a promising metric to potentially screen ADHD symptoms amongst children across different age range.

Our findings from this study initially suggested that age was a major contributor to differences in the performance on the eye tracking tasks, with the younger participants generally having a shorter fixation duration, more intrusive saccades, a lower adjusted accuracy, and a longer correction latency. The simple fixation task and antisaccade trials rely on focusing attention and having inhibitory control over saccades that require the frontal cortex (22, 23). Given that older children have a slightly more matured frontal cortex, it was expected that the older children in this study would perform better on these measures. This is also consistent with previous literature reports demonstrating that the number of intrusive saccades during simple fixation is reduced as children age and that their performance on antisaccade improves after the developmental age of 10 years old (24, 25).

The diagnosis was a factor that influenced differences on the eye tracking tasks. In particular, the ADHD group, with inattention, hyperactivity, and impulsivity tendencies, had a shorter fixation duration and more intrusive saccades, made more errors, and required more time to correct erroneous saccades. These findings also have been observed in other studies in which participants with ADHD incurred more errors during antisaccade trials, which can persist into adulthood. The poorer performance of the ADHD participants during the antisaccade trials compared to the prosaccade trials also suggests that frontal cortex inhibition control in these participants was less developed (26). Altogether, this result supports the notion that it might be possible to utilize the simple fixation and antisaccade eye tracking tasks to identify ADHD-like symptoms by measuring the fixation duration, the number of saccades during simple fixation, the number of errors, and the latency to correct erroneous saccades.

Finally, this study also demonstrated the robustness of the AI model that we developed for the test, which was able to successfully discriminate the eye movements of the TD and ADHD groups. As it is well known that the robustness of an AI model highly depends on the training data it is provided, there is always room for improvements for our current AI model (27). To increase the generalizability of the model and to enhance its robustness in tolerating changes in variance within a group of children, the data should be trained on a dataset comprising of children across different age groups. Such an approach will ensure that the model is not biased toward a specific age group and will enable it to perform better on a wider range of data. By using a diverse dataset, the model will also be able to recognize patterns and features that are common across different age groups and will be less prone to overfitting. Ultimately this will lead to a more accurate and reliable gaze estimation.

Nevertheless, there are some limitations to our study that must be addressed. For example, our results might have been due to the short fixation time (30 s) and the limited number of trials (14 prosaccade and 14 antisaccade trials), which might not have been long enough to detect significant changes. Additionally, we included participants who had completed at least three prosaccade and three antisaccade trials, which might not have been sufficient for analyses. An achievable solution would be to sample at least 25 successful trials, as demonstrated by other researchers (28). In addition, a future experiment should be performed to determine whether the same parameters can differentiate the TD and those with ADHD subtypes such as combination ADHD, hyperactive/impulsive ADHD, and inattentive ADHD with more participants.

As this is a task that required both attention and judgment, variation dependent on the test time may result in variations in performance. These differences could stem from physical factors, such as fatigue during the day after exercise, or mental factors, such as mental exhaustion after a long day of class. Hunger resulting from approaching lunch time may also affect performance. Although the actual test only lasts 6 min, administrative and logistic factors may contribute to a longer test time. As such, performing tests at a stipulated time window of the day may be useful for future studies. Finally, as the parameters of the stimuli used in the eye tracking test have been optimized for a 13-inch tablet, we plan to refine the test algorithm to allow it to be administered on screens of other dimensions.

Here, we successfully developed AI-based eye tracking technology that can be operated on a tablet, without the need for sophisticated equipment, to detect ADHD symptoms in individuals. In China, where accessibility to professional pediatric psychiatric services is limited, this technology can be easily implemented in schools to identify students at risk of ADHD. Importantly, the results provided are objectively quantifiable, thus allowing students to receive intervention earlier and additional help to cope in school. Moreover, it would allow parents to understand their child better and improve their quality of life. Besides its utility in screening children, it would be worthwhile to explore in future studies whether it is a useful tool for objectively assessing if a child’s ADHD symptoms have improved after introducing either behavioral or pharmacological interventions.

## Data availability statement

The raw data supporting the conclusions of this article will be made available by the authors, without undue reservation.

## Ethics statement

The studies involving humans were approved by the Medical Ethics Committee of West China Second University Hospital of Sichuan University. The studies were conducted in accordance with the local legislation and institutional requirements. The participants provided their written informed consent to participate in this study.

## Author contributions

XC: Conceptualization, Data curation, Funding acquisition, Writing – original draft. SW: Data curation, Formal analysis, Methodology, Writing – original draft. XY: Data curation, Formal analysis, Methodology, Writing – original draft. CY: Data curation, Formal analysis, Methodology, Writing – original draft. FN: Data curation, Formal analysis, Methodology, Writing – original draft. JYa: Data curation, Formal analysis, Methodology, Writing – original draft. YT: Data curation, Formal analysis, Methodology, Writing – original draft. JYe: Formal analysis, Methodology, Writing – review and editing. HL: Formal analysis, Methodology, Writing – review and editing. RL: Conceptualization, Funding acquisition, Project administration, Supervision, Writing – review and editing.

## Supplementary material

Supplementary Figure 1 The two distinct tasks are illustrated here: **(A)** the simple fixation task, and **(B)** the prosaccade/antisaccade task. A hybrid approach was implemented by combining the Gap paradigm and the Overlap paradigm. The task consisted of 28 trials, counterbalanced between 14 prosaccade (seven with target on the left) and 14 antisaccade (seven with target on the left) conditions. Participants were presented the trials in a randomized order. The Gap denoted the 200 ms period following the initial fixation, while the Overlap involved a central fixation point appearing alongside the lateral target. The differentiation between the prosaccade and antisaccade tasks lied in the eye movement response relative to the lateral target based on the color of the central point. A green dot indicated a prosaccade task, and the red dot indicated an antisaccade task. This is a royalty-free image obtained from https://www.freeimages.com/photo/handsome-male-traveler-1637362.

Supplementary Figure 2 Overview of our eye tracking CNN. Inputs include right eye, left eye, together with a face image clipped from the original frame. A 1 × 6 vector represents the coordination of the locations of the eyes and face. The output is the predicted gaze location in pixels. ImageNet: ImageNet Large-Scale Visual Recognition Challenge (ILSVRC) is an annual event to showcase and challenge computer vision models. VGG16: VGG16 is a type of CNN with 16–19 weight layers (approximately 138 trainable parameters). VGG16 structure contains Fully Connected Layers, but here we only used the convolution layers of VGG16.

Supplementary Figure 3 The AI model used was implemented based on the application program on the tablet. The Relay Centre in the figure represents the eye movement assessment software installed on the Lenovo Yoga 13-inch tablet, serving as a relay station for both efferent (blue lines) information, including presenting visual stimuli information to the subject and transmitting video and task parameter information to the cloud, and afferent (red lines) information, including receiving facial feature video information of the subject’s gaze point and eye movement parameter result information analyzed by the AI model.
