# Supplementary figures and images for: Utilizing artificial intelligence-based eye tracking technology for screening ADHD symptoms in children
Source: Front Psychiatry. 2023 Nov 14;14:1260031. doi: 10.3389/fpsyt.2023.1260031 (PMC10682190; doi:10.3389/fpsyt.2023.1260031)

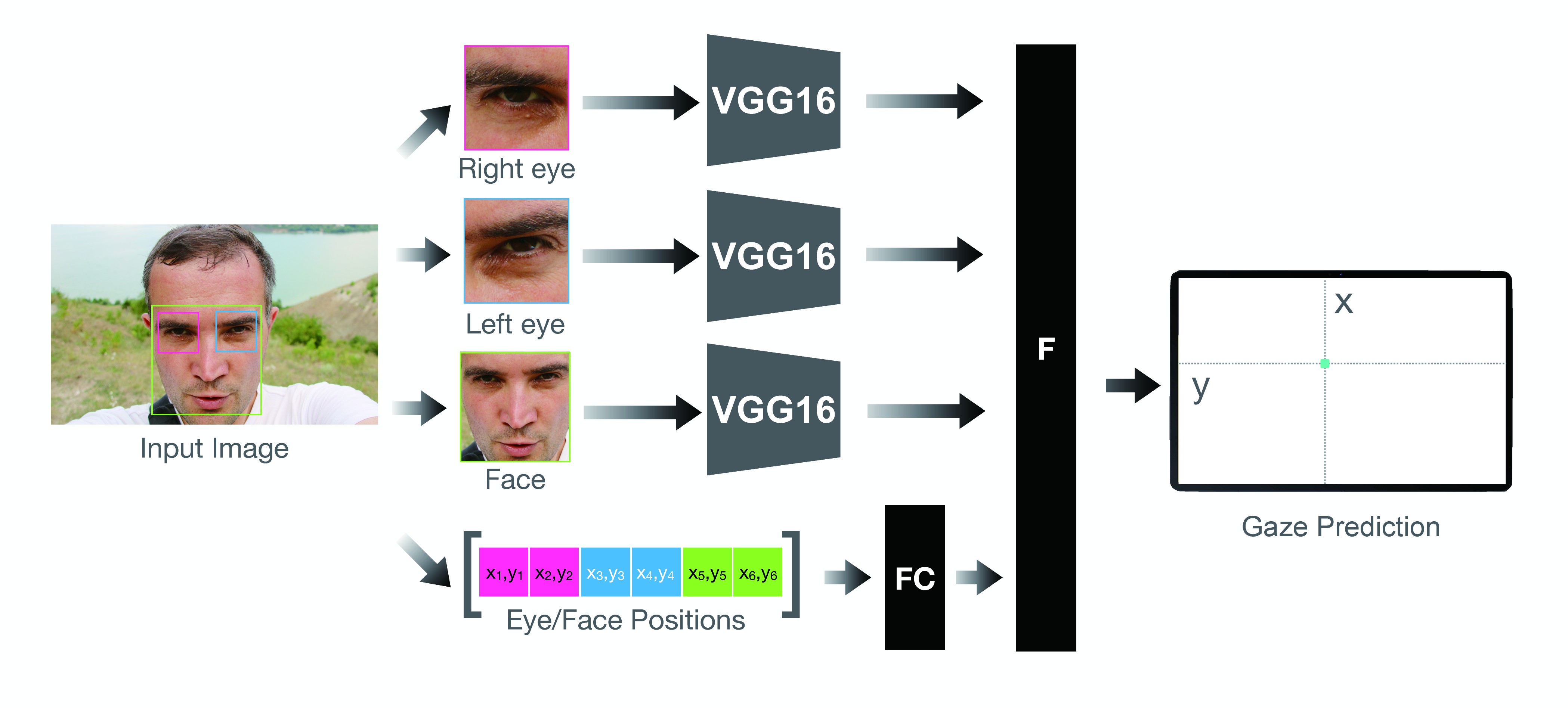

Supplement: Supplementary Figure 1 — The two distinct tasks are illustrated here: (A) the simple fixation task, and (B) the prosaccade/antisaccade task. A hybrid approach was implemented by combining the Gap paradigm and the Overlap paradigm. The task consisted of 28 trials, counterbalanced between 14 prosaccade (seven with target on the left) and 14 antisaccade (seven with target on the left) conditions. Participants were presented the trials in a randomized order. The Gap denoted the 200 ms period following the initial fixation, while the Overlap involved a central fixation point appearing alongside the lateral target. The differentiation between the prosaccade and antisaccade tasks lied in the eye movement response relative to the lateral target based on the color of the central point. A green dot indicated a prosaccade task, and the red dot indicated an antisaccade task. This is a royalty-free image obtained from https://www.freeimages.com/photo/handsome-male-traveler-1637362. [file Image_1.TIF]

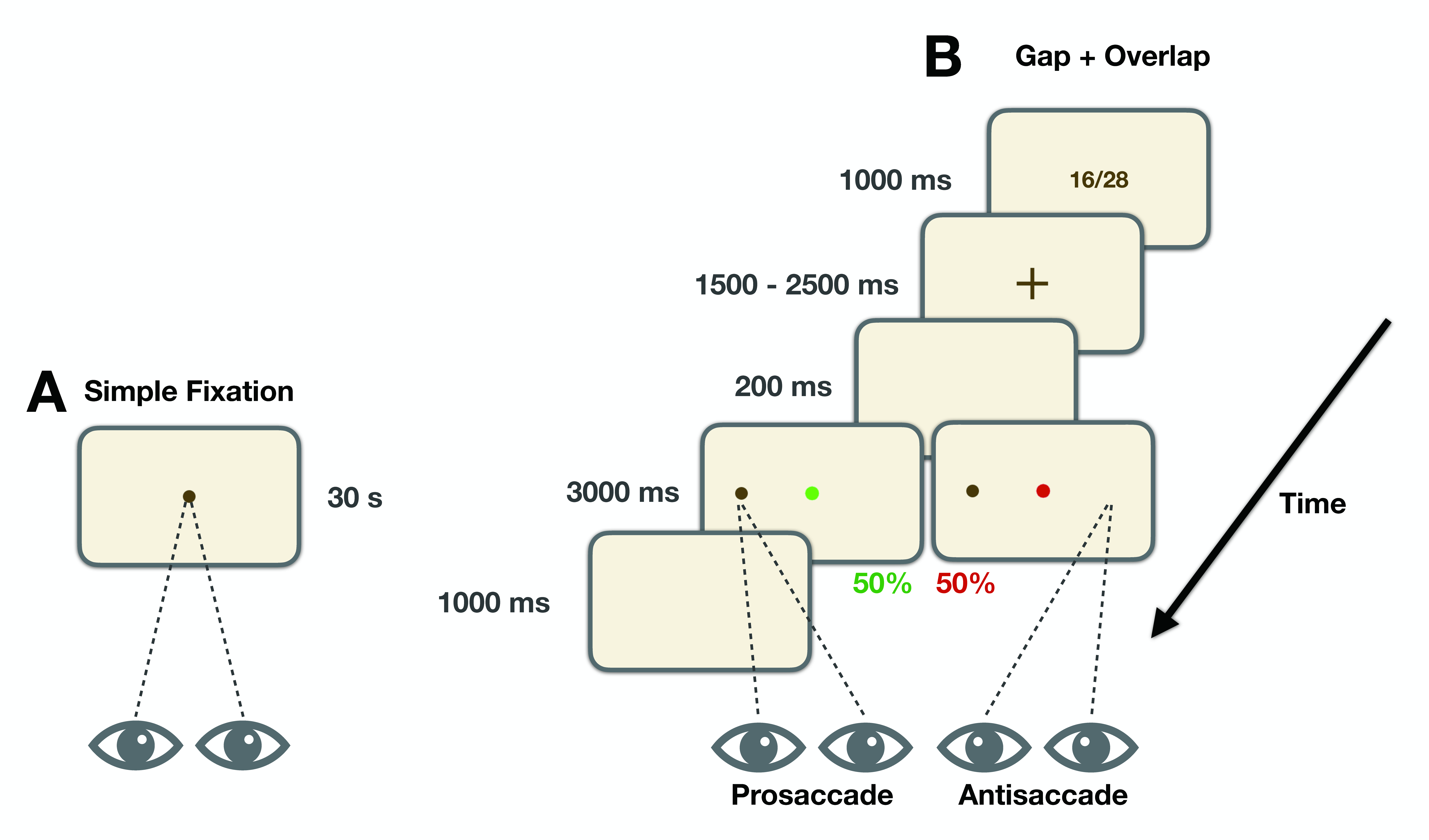

Supplement: Supplementary Figure 2 — Overview of our eye tracking CNN. Inputs include right eye, left eye, together with a face image clipped from the original frame. A 1 × 6 vector represents the coordination of the locations of the eyes and face. The output is the predicted gaze location in pixels. ImageNet: ImageNet Large-Scale Visual Recognition Challenge (ILSVRC) is an annual event to showcase and challenge computer vision models. VGG16: VGG16 is a type of CNN with 16–19 weight layers (approximately 138 trainable parameters). VGG16 structure contains Fully Connected Layers, but here we only used the convolution layers of VGG16. [file Image_2.TIF]

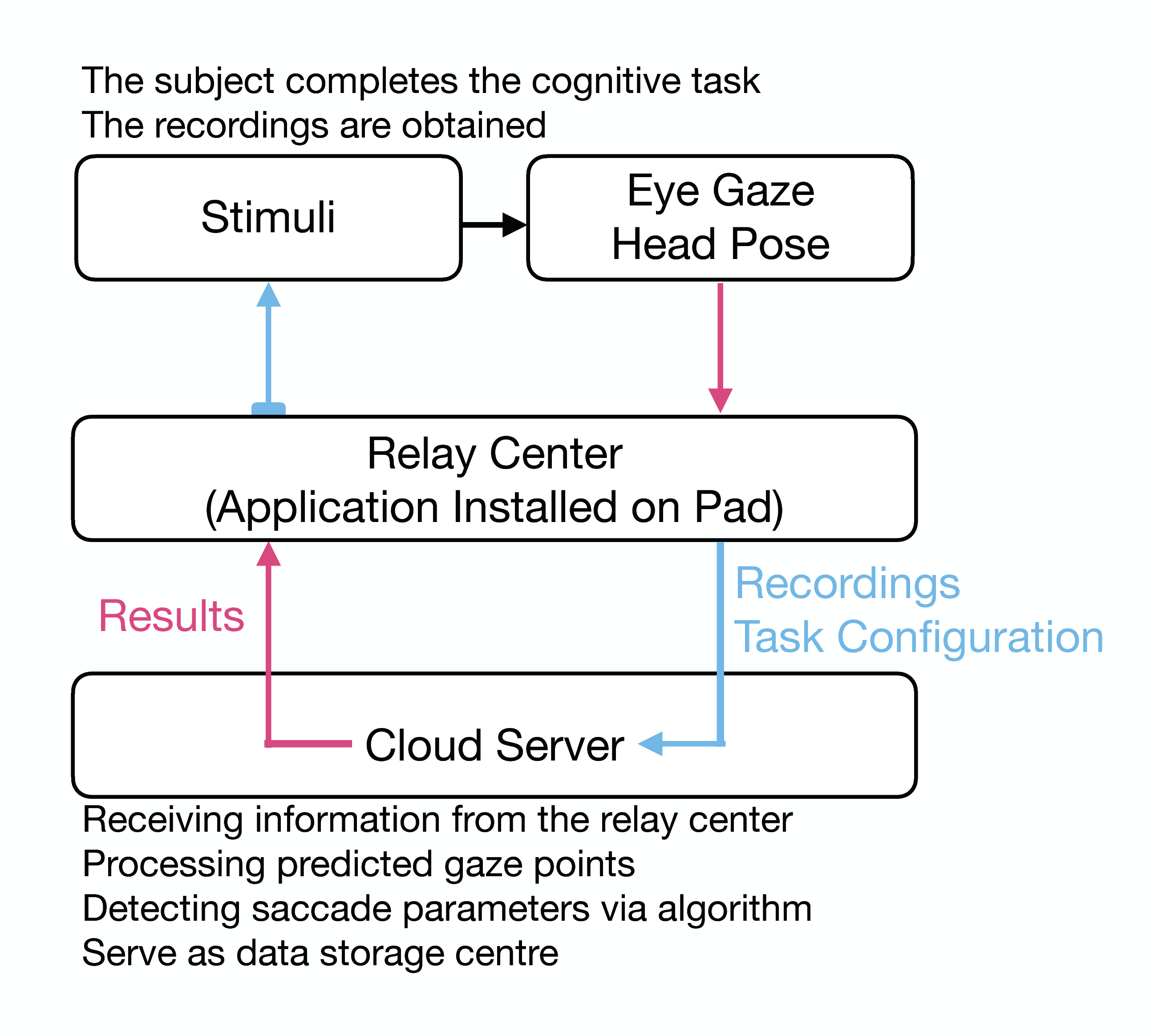

Supplement: Supplementary Figure 3 — The AI model used was implemented based on the application program on the tablet. The Relay Centre in the figure represents the eye movement assessment software installed on the Lenovo Yoga 13-inch tablet, serving as a relay station for both efferent (blue lines) information, including presenting visual stimuli information to the subject and transmitting video and task parameter information to the cloud, and afferent (red lines) information, including receiving facial feature video information of the subject’s gaze point and eye movement parameter result information analyzed by the AI model. [file Image_3.TIF]
